# A Novel Copper Ionophore Nanoshuttle (Winged Cu) for Inducing Cuproptosis in B16 Melanoma Cells

**DOI:** 10.3390/biom15060895

**Published:** 2025-06-18

**Authors:** Yuhuan Wu, Ziyao Chang, Wenhao Wang, Chuanbin Wu, Xin Pan, Zhengwei Huang

**Affiliations:** 1 School of Pharmaceutical Science, Sun Yat-Sen University, Guangzhou 510006, China; wuyh83@mail2.sysu.edu.cn (Y.W.); changzy@mail2.sysu.edu.cn (Z.C.); wangwh37@mail2.sysu.edu.cn (W.W.); 2 State Key Laboratory of Bioactive Molecules and Druggability Assessment, Guangdong Basic Research Center of Excellence for Natural Bioactive Molecules and Discovery of Innovative Drugs, College of Pharmacy, Jinan University, Guangzhou 510632, China; chuanbinwu@jnu.edu.cn

**Keywords:** cuproptosis, copper ionophore, intracellular copper accumulation, nanoshuttle, B16 melanoma cells

## Abstract

Cuproptosis, a newly discovered copper-dependent programmed cell death pathway, represents a promising approach for anticancer therapy. However, the efficacy of cuproptosis critically depends on intracellular copper accumulation. Traditional copper ionophores have limited therapeutic efficacy due to their reliance on serum copper levels. Therefore, the development of novel copper ionophores to enhance intracellular copper levels is urgently needed. In this study, we targeted a melanoma model and pioneered the application of Bis(2-hydroxyethyl)dithiocarbamic acid copper(II) [Cu(HEDTC)_2_] as a highly efficient copper ionophore for inducing cuproptosis in B16 melanoma cells. Compared to conventional copper ionophores, Cu(HEDTC)_2_ exhibits superior intracellular copper delivery efficiency, thereby enhancing the induction of cuproptosis. We further constructed a Cu(HEDTC)_2_@Soluplus-nanomicelle (CS NM) system designed to disrupt copper ion homeostasis in tumor cells and amplify cuproptosis. In this system, Cu(HEDTC)_2_, as a novel copper ionophore, significantly enhanced the copper level in B16 melanoma cells. Upon cellular internalization, CS NM underwent degradation and released copper ions, which subsequently triggered cuproptosis by causing abnormal aggregation of mitochondrial lipoylated proteins. This study provides a new experimental foundation and potential therapeutic strategy for cuproptosis-based cancer treatment.

## 1. Introduction

Cuproptosis, as a burgeoning form of programmed cell death, has garnered significant interest in cancer therapeutics [1]. Distinct from other well-characterized forms of programmed cell death, such as apoptosis, necrosis, pyroptosis, and ferroptosis, cuproptosis is uniquely dependent on the sustained intracellular accumulation of copper ions [2]. Mechanistically, copper directly binds to lipoylated proteins in the tricarboxylic acid (TCA) cycle, particularly dihydrolipoamide S-acetyltransferase (DLAT), triggering abnormal aggregation of mitochondrial lipoylated proteins and the depletion of iron–sulfur (Fe-S) cluster proteins [3]. These events ultimately induce proteotoxic stress and irreversible cell death [3]. As such, it has been well documented that cuproptosis-based therapy is a promising anticancer strategy. Pharmacological studies have demonstrated that excessive intracellular copper ions can induce cuproptosis, effectively eliminating various cancer cell lines, including melanoma and ovarian carcinoma [4,5].

Despite its therapeutic potential, the clinical translation of cuproptosis-based anticancer therapy still faces considerable challenges [6]. Most cancer cells maintain strict copper homeostasis at low levels through sophisticated regulatory mechanisms, which restrict the intracellular accumulation of exogenous copper ions, thereby undermining the efficacy of cuproptosis-based therapy [7]. Consequently, developing strategies to effectively disrupt copper ion homeostasis in tumor cells and promote intracellular copper accumulation represents a critical frontier in cuproptosis-based anticancer therapy.

Currently, various copper ionophores have been developed to transport copper ions for the disruption of tumoral copper ion homeostasis. The extensively studied copper ionophores such as elesclomol [8,9,10] and disulfiram (DSF) [8,11] exhibit significant limitations in practical applications [12]. Primarily, these copper ionophores do not intrinsically contain copper ions but rather rely on binding to serum copper to form active complexes for tumor uptake [13]. Consequently, their therapeutic efficacy is highly dependent on patients’ serum copper levels, with markedly reduced antitumor effects observed in patients with hypocupremia [14]. Furthermore, these copper-free ionophores may interfere with the copper metabolism in normal cells, leading to nonspecific toxicity [11]. Plausible alternative strategies have been explored for direct copper ion delivery [15]. However, this approach poses even greater challenges. Free copper ions demonstrate poor bioavailability in vivo, making it difficult to achieve effective concentrations at tumor sites through direct delivery [16]. Moreover, the widespread distribution of copper ions may cause systemic side effects [17]. These critical issues underscore the urgent need to develop a “dynamic duo” system, i.e., novel copper ionophores that contain copper ions, to overcome the individual disadvantages of copper-free ionophores and pristine copper ions.

**Scheme 1:**
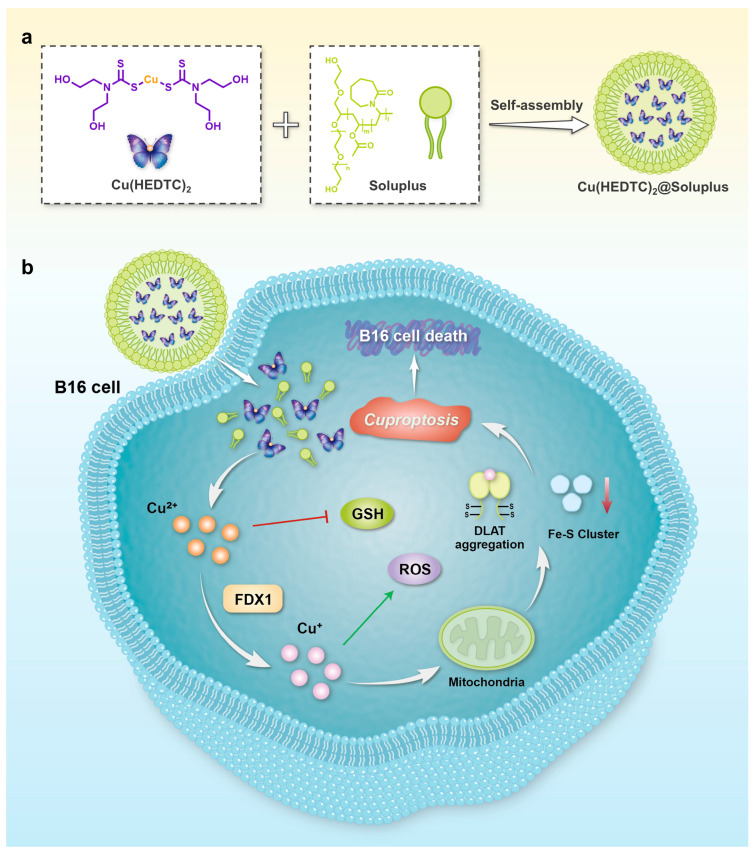
Illustration of the strategy of CS NM-based cuproptosis therapy. (**a**) Schematic illustration showing the preparation of CS NM. (**b**) Biological mechanisms of cuproptosis in B16 cell induced by CS NM. Upon cellular internalization, CS NM releases Cu(HEDTC)_2_ into B16 cells, subsequently dissociating copper ions. These ions deplete intracellular glutathione (GSH), promote the accumulation of reactive oxygen species (ROS), and induce oxidative stress, thereby facilitating cuproptosis. Meanwhile, they directly bind to mitochondrial lipoylated dihydrolipoamide S-acetyltransferase (DLAT), inducing its abnormal aggregation and downregulation of Fe-S cluster proteins, ultimately triggering cuproptosis.

Herein, confronting the clinical demands of melanoma management, a cancer that accounts for over 80% of skin cancer deaths [18], we present the first proposal and demonstration of Bis(2-hydroxyethyl)dithiocarbamic acid copper(II) (denoted as Cu(HEDTC)_2_) as a novel copper ionophore with cancer therapeutic potential. To optimize its delivery efficiency, we further developed a “Winged Cu” nanoshuttle system, a Soluplus-based nanomicelle system encapsulating Cu(HEDTC)_2_ (Winged Cu), to induce cuproptosis in B16 murine melanoma cells (model melanoma cell) through copper homeostasis disruption strategies. On one hand, Cu(HEDTC)_2_, a stable copper complex with wing-like ligand conformation, serves as an ideal copper ionophore that can directly provide and protect exogenous copper ions. On the other hand, Soluplus, an FDA-approved pharmaceutical excipient, is a triblock copolymer composed of polyethylene glycol (PEG), polyvinyl caprolactam (PVCL), and polyvinyl acetate (PVAc) [19]. Its unique amphiphilic structure enables the formation of stable nanomicelles that effectively encapsulate hydrophobic Cu(HEDTC)_2_ [20], yielding Cu(HEDTC)_2_@Soluplus nanomicelles (denoted as CS NM, Scheme 1a). Fabrication of CS NM not only enhances the aqueous solubility and bioavailability of Cu(HEDTC)_2_ [20,21] but also facilitates tumor-specific accumulation via the enhanced permeability and retention (EPR) effect [22], functioning as a nanoshuttle for intracellular copper delivery. Upon cellular internalization, the CS NM nanoshuttle releases Cu(HEDTC)_2_, which subsequently dissociates to copper ions. The liberated copper ions deplete the glutathione (GSH) within the tumor cells and promote the accumulation of reactive oxygen species (ROS), inducing oxidative stress, which facilitates cuproptosis. Additionally, they directly bind to lipoylated DLAT protein in mitochondria, leading to aberrant protein aggregation and the downregulation of Fe-S cluster proteins, ultimately triggering cuproptosis (Scheme 1b). This strategy endows CS NM with superior efficacy in inducing B16 cell death, establishing a promising new strategy for cuproptosis-based melanoma therapy. Therefore, this study aimed to develop a novel copper ionophore-based nanosystem and investigate its mechanism for inducing cuproptosis for effective melanoma treatment. To highlight the distinctiveness of CS NM and address the limitations of previous studies, we have summarized the key differences in Table 1.

## 2. Materials and Methods

### 2.1. Materials

All reagents used were of analytical grade. Soluplus^®^ was kindly provided by BASF Ltd. (Shanghai, China). Bis(2-hydroxyethyl)dithiocarbamic acid copper(II) (Cu(HEDTC)2), tetrathiomolybdate (TTM) and 2-cyano-3-(1-phenylindol-3-yl)acrylate (UK-5099) were obtained from Macklin Biochemical Co., Ltd. (Shanghai, China). N-Acetyl-L-Cysteine (NAC) was obtained from Aladdin Biochemical Technology Co., Ltd. (Shanghai, China). Ethanol absolute was obtained from Damao Chemical Reagent Factory (Tianjin, China). RPMI 1640 medium, Phosphate-buffered saline (PBS), fetal bovine serum (FBS), 0.25% EDTA-Trypsin and BCA Protein Assay were provided by Thermo Fisher (Shanghai, China). Cell counting kit-8 was purchased from DIB Data Inventory Biotechnology (Guangzhou, China). RIPA lysate was purchased from Beyotime Biotechnology (Shanghai, China). Coumarin 6 (C6) was purchased from MACLIN Ltd. (Shanghai, China). DAPI and DCFH-DA were purchased from Solarbio Life Science (Beijing, China). GSH level assay kit was purchased from Nanjing Jiancheng Bioengineering Institute (Nanjing, China). Cell copper assay kit was purchased from Elabscience (Wuhan, China). DLAT antibody (Catalog: 13426-1-AP) and LIAS antibody (Catalog: 11577-1-AP) purchased from Proteintech (Wuhan, China). FDX1 antibody (Catalog: T510671S) was purchased from Abmart (Shanghai, China). ATP7A antibody (Catalog: DF8506) and Goat Anti-Rabbit lgG (H+L) Fluor488-conjugated antibody (Catalog: S0018) were purchased from Affinity Biosciences (Beijing, China).

### 2.2. Soluplus Concentration Confirmation

Soluplus was dissolved in distilled water to different concentrations. Then, the Soluplus solution was stirred at 450 rpm for 24 h to obtain a series of concentrations of Soluplus-Mil (20, 30, 40, 50, and 60 mg/mL). Finally, the Soluplus-Mil was collected, and its mean particle size, polydispersity index (PDI), and zeta potential were detected by the Malvern Zetasizer (Nano ZS90, Malvern Instruments Ltd., Worcestershir, UK). Each sample was analyzed in triplicate (*n* = 3) and the results are presented as the mean ± standard deviation (SD).

### 2.3. Synthesis of Cu(HEDTC)_2_@Soluplus Nanomicelles

The Cu(HEDTC)_2_-Mil (CS NM) were prepared by the thin-film dispersion method [26]. Briefly, Cu(HEDTC)_2_ was dissolved into 1 mL ethanol to 500 μM. Then, 3 mL of Cu(HEDTC)_2_ solution and 40 mg of Soluplus were mixed in a penicillin bottle and stirred at 450 rpm for 30 min at room temperature. After mixing, the penicillin bottle was transferred to a vacuum-drying oven (BPZ-6210-2, Shanghai, China) for complete evaporation of ethanol. Finally, 1 mL distilled water was added under stirring at 500 rpm for 24 h at room temperature to obtain CS NM with a concentration of 1.5 mM (total Cu(HEDTC)_2_ in 1 mL distilled water).

### 2.4. Characterization of Cu(HEDTC)_2_@Soluplus Nanomicelles

The particle size, polydispersity index (PDI) and zeta potential of Cu(HEDTC)_2_-Mil (CS NM) were measured by the Malvern Zetasizer (Nano ZS90, Malvern Instruments Ltd., Worcestershir, UK). Each sample was analyzed in triplicate (*n* = 3). The morphology of CS NM was measured via transmission electron microscopy (TEM) (JEM1400, Hitachi, Japan). The UV–Vis measurements were performed as follows: Soluplus and CS NM were dissolved in 2 mL ultrapure water with ultrapure water as reference baseline, while Cu(HEDTC)_2_ was dissolved in ethanol with ethanol reference baseline for qualitative analysis. For quantitative analysis, Cu(HEDTC)_2_ standard solutions were prepared in anhydrous ethanol at different concentrations (18.75, 25, 30, 37.5, 50, and 60 μM), with ethanol serving as the reference baseline. Each Cu(HEDTC)_2_ concentration was prepared in triplicate (*n* = 3), and the results were recorded to establish the standard calibration curve. All UV–Vis absorption spectra were recorded from 200 to 800 nm using an ultraviolet spectrometer (TU-1901, Purkinje General Instrument Co., Ltd., Beijing, China) with 1 cm path-length quartz cuvettes. Fourier transform infrared (FTIR) spectroscopy was conducted on an FTIR spectrometer (UATR Two, PerkinElmer, Waltham, MD, USA) with wavenumbers ranging from 400 to 4000 cm^−1^. CS NM samples were lyophilized and directly analyzed by attenuated total reflectance (ATR)-FTIR, whereas solid-state Soluplus and Cu(HEDTC)_2_ were pressed onto the ATR crystal without pretreatment. All measurements were performed at 25 °C.

### 2.5. Intracellular Cu Content Assay

Melanoma cells (B16 cells) were purchased from Procell Life Science & Technology Co., Ltd. (Wuhan, China) [27]. To investigate the Cu content in CS NM-treated cells, B16 cells were seeded in 10 cm cell culture dishes at a density of 3 × 10^6^ per dish in RPMI 1640 complete medium (10% FBS, 1% penicillin/streptomycin), and cultured for 24 h. Then, the cells were treated with RPMI 1640 complete medium containing different concentrations of CS NM (0, 10, 20, and 30 μM) for 24 h. Then, the cells were washed with PBS three times. After PBS washing, the cells were lysed in RIPA lysate (containing 1% protease inhibitor) on ice for 10 min. The lysates were centrifuged at 12,000 rpm for 10 min at 4 °C, and the supernatants were harvested for copper and protein quantification. Then, the intracellular Cu content was determined following the instructions for the cell copper assay kit (Elabscience, Wuhan, China), and normalized to the total protein concentration determined by the BCA Protein Assay (Thermo Fisher, Shanghai, China). The absorbance value was measured at 580 nm using a microplate reader. Results are presented as the mean ± standard deviation (SD) of three independent experiments (*n* = 3).

### 2.6. Cellular Uptake

The cellular uptake was detected by the Coumarin 6 (C6). B16 cells were seeded in a 20 mm glass-bottom cell culture dish at a density of 6 × 10^5^ per dish in RPMI 1640 complete medium and cultured for 24 h. Then, the cells were treated with RPMI 1640 complete medium containing C6-Mil (1 μg mL^−1^) for 1 h, 2 h and 4 h. Then, the cells were stained with DAPI for 10 min. The cells were observed and imaged by the confocal laser scanning microscope (CLSM). For quantitative analysis, B16 cells were seeded in the 6-well plate at a density of 6 × 10^5^ per well and cultured for 24 h. Then, the cells were treated with C6-Mil (1 μg mL^−1^) for 1 h, 2 h and 4 h. Then, the cells were digested with 0.25% EDTA-Trypsin, and the cells were collected and gently washed with PBS three times. The cells were collected and subjected to flow cytometry (CytoFLEX S, Beckmann). The raw fluorescence data were normalized to cell count using ImageJ v1.53 (National Institutes of Health, Bethesda, MD, USA) by quantifying viable cell counts from bright-field images of each sample prior to analysis. Mean fluorescence intensity (MFI) values were calculated and expressed as MFI ± standard deviation (SD). Statistical significance was determined by one-way ANOVA using GraphPad Prism 9.0 (GraphPad Software LLC., San Diego, CA, USA). Results are presented as the mean ± standard deviation (SD) of three independent experiments (*n* = 3).

### 2.7. Cytotoxicity Assay

B16 cells were seeded in a 96-well plate at a density of 1.2 × 10^4^ per well in RPMI 1640 complete medium and cultured for 24 h. Then, the cells were treated with RPMI 1640 complete medium containing different concentrations of CuCl_2_, CS NM, and Cu(HEDTC)_2_ to assess comparative cytotoxicity (0, 5, 10, 15, 20, 25, and 30 μM, Cu-equivalent concentration). Corresponding control groups included DMSO solvent (0, 017, 0.33, 0.5, 0.67, 0.83, and 1% *v*/*v*, matching Cu(HEDTC)_2_ concentrations) and Soluplus nanomicelles (0, 133, 267, 400, 533, 667, and 800 μg/mL, matching CS NM concentrations) to evaluate cell biocompatibility. After another 24 h, the cell viability was detected using CCK-8 assay. The absorbance value was measured at 450 nm using a microplate reader. The IC_50_ values were calculated by fitting the dose–response curve using nonlinear regression (log(inhibitor) vs. normalized response–variable slope) in GraphPad Prism 9.0 (GraphPad Software LLC., San Diego, CA, USA). Each sample was prepared and tested in sextuplicate (*n* = 6), and the results are presented as the mean ± standard deviation (SD).

### 2.8. Biocompatibility Test

RAW 264.7 cells were donated by Prof. Feng Min’s group from Sun Yat-sen University (Guangzhou, China). Human umbilical vein endothelial cells (HUVECs) were donated by Prof. Xu Yuehong’s group from Sun Yat-sen University (Guangzhou, China). For the biocompatibility test, the biocompatibility of Soluplus was tested in RAW 264.7 cells and HUVECs. RAW 264.7 cells and HUVECs were seeded in a 96-well plate at a density of 1 × 10^4^ per well in RPMI 1640 complete medium and cultured for 24 h. Then, the cells were treated with RPMI 1640 complete medium containing different concentrations of Soluplus (0, 200, 400, 600, 800, 1000, and 1200 μg/mL). After another 24 h, the cell viability was detected using CCK-8 assay. The absorbance value was measured at 450 nm using a microplate reader. Each sample was prepared and tested in sextuplicate (*n* = 6), and the results are presented as the mean ± standard deviation (SD).

### 2.9. Antioxidant Rescue Assay

To investigate whether CS NM-induced cytotoxicity is mediated through ROS/GSH, the antioxidant N-Acetyl-L-Cysteine (NAC) (50 μM) was employed to rescue B16 cells from CS NM cytotoxicity. Briefly, B16 cells were seeded in a 96-well plate at a density of 1 × 10^4^ per well in RPMI 1640 complete medium and cultured for 24 h. Then, the cells were treated with RPMI 1640 complete medium containing NAC (50 μM). After another 24 h, the cell viability was detected using CCK-8 assay. The absorbance value was measured at 450 nm using a microplate reader. Each sample was prepared and tested in triplicate (*n* = 3), and the results are presented as the mean ± standard deviation (SD).

### 2.10. Intracellular GSH Level Detection

To investigate the GSH level in CS NM-treated cells, B16 cells were seeded in 6-well plates at a density of 6 × 10^5^ per well and cultured for 24 h. Then, the cells were treated with RPMI 1640 complete medium containing different concentrations of CS NM (0, 10, 20, and 30 μM) for 24 h. Then, the cells were washed with PBS three times and lysed in RIPA lysate (containing 1% protease inhibitor) on ice for 10 min. The cell lysate was obtained by centrifugation at 4 °C, 12000× *g* for 10 min. Then, the intracellular GSH level was determined following the instructions for the GSH level assay kit (Nanjing Jiancheng Bioengineering Institute, Nanjing, China), and normalized to the total protein concentration determined by the BCA Protein Assay (Thermo Fisher, Shanghai, China). The absorbance value was measured at 405 nm using a microplate reader. Results are presented as the mean ± standard deviation (SD) of three independent experiments (*n* = 3).

### 2.11. Intracellular ROS Generation Assay

The intracellular ROS was detected by a specific probe DCFH-DA [28]. B16 cells were seeded in a 20 mm glass-bottom cell culture dish at a density of 6 × 10^5^ per dish in RPMI 1640 complete medium and cultured for 24 h. Then, the cells were treated with RPMI 1640 complete medium containing different concentrations of CS NM (0, 7.5, 15 and 30 μM) for 4 h. The cells were stained with DCFH-DA for 30 min. The cells were observed and imaged using the CLSM. For quantitative analysis, B16 cells were seeded in the 6-well plate at a density of 6 × 10^5^ per well and cultured for 24 h. Then, the cells were treated with RPMI 1640 complete medium containing different concentrations of CS NM (0, 7.5, 15 and 30 μM) for 4 h. Then, the cells were gently washed with PBS three times and stained with DCFH-DA. After staining for 30 min, the cells were collected and subjected to flow cytometry (CytoFLEX S, Beckmann). The raw fluorescence data were normalized to cell count using ImageJ v1.53 (National Institutes of Health, Bethesda, MD, USA) by quantifying viable cell counts from bright-field images of each sample prior to analysis. Mean fluorescence intensity (MFI) values were calculated and expressed as MFI ± standard deviation (SD). Statistical significance was determined by one-way ANOVA using GraphPad Prism 9.0 (GraphPad Software LLC., San Diego, CA, USA). Results are presented as the mean ± standard deviation (SD) of three independent experiments (*n* = 3).

### 2.12. Cell Death Form Confirmation

To study whether CS NM has a cuproptosis pathway that induces B16 cell death, two cuproptosis inhibitors, viz. 2-cyano-3-(1-phenylindol-3-yl)acrylate (UK-5099) (10 μM) and Tetrathiomolybdate (TTM) (10 μM) were employed for the CS NM to measure the cell viability by CCK-8 assay [29]. Briefly, B16 cells were seeded in a 96-well plate at a density of 1.2 × 10^4^ per well in RPMI 1640 complete medium and cultured for 24 h. Then, the cells were treated with RPMI 1640 complete medium containing UK-5099 (10 μM) and TTM (10 μM), respectively. After another 24 h, the cell viability was detected using CCK-8 assay. The absorbance value was measured at 450 nm using a microplate reader. Each sample was prepared and tested in triplicate (*n* = 3), and the results are presented as the mean ± standard deviation (SD).

### 2.13. Cell Mitochondrial Morphology

B16 cells were seeded in a 6 cm cell culture dish at a density of 1 × 10^6^ per dish and cultured for 24 h. Then, the cells were treated with RPMI 1640 complete medium containing CS NM (15 μM) for another 24 h. Then, the cells were washed and harvested gently, then fixed with 2.5% glutaraldehyde. The samples were subjected to biological transmission electron microscopy (Bio-TEM) (HT7800/HT7700, HITACHI, Tokyo, Japan).

### 2.14. Immunofluorescence of DLAT Oligomerization and FDX1, LIAS and ATP7A Expression

B16 cells were seeded in a 20 mm glass-bottom cell culture dish at a density of 6 × 10^5^ per dish in RPMI 1640 complete medium and cultured for 24 h. Then, the cells were treated with RPMI 1640 complete medium containing different concentrations of CS NM (0, 5, 10, and 20 μM) for another 24 h. The cells were fixed with 4% PFA for 10 min. Then, the fixed cells were incubated with the DLAT antibody (dilution 1:250), FDX1 antibody (dilution 1:200), LIAS antibody (dilution 1:500) and ATP7A antibody (dilution 1:300) at 4 °C overnight, respectively. After incubation, the cells were incubated with an Alexa Fluor 488-conjugated anti-rabbit secondary antibody at room temperature for 1 h. Then, the cells were stained with DAPI at room temperature for 10 min and detected by CLSM. Each sample was analyzed in triplicate (*n* = 3).

### 2.15. Western Blot

The Western blot method was utilized to analyze the specific protein expression level. B16 cells were seeded in a 6 cm cell culture dish at a density of 1 × 10^6^ per dish in RPMI 1640 complete medium and cultured for 24 h. Then, the cells were treated with RPMI 1640 complete medium containing CS NM (15 μM) for another 24 h. Then, the cells were washed with PBS three times and lysed completely. The cell lysate was centrifuged at 4 °C, 12,000× *g* for 10 min to obtain the cell proteins. Then, the cell proteins were subjected to Western blot analysis. The protein bands were quantified using ImageJ v1.53 (National Institutes of Health, Bethesda, MD, USA). Each sample was analyzed in three independent experiments (*n* = 3).

### 2.16. Statistical Analysis

All data are expressed as mean ± standard deviation (SD) [30]. The specific statistical sample size for each experiment is shown in the figure legend. Statistical analyses were evaluated using one-way analysis of variance (ANOVA) by GraphPad Prism (version 9.0, GraphPad Software LLC., San Diego, CA, USA), while two-group comparisons were performed using unpaired *t*-tests. *p* < 0.05 was considered statistically significant, and *p* < 0.01, *p* < 0.001 and *p* < 0.0001 were considered to indicate high significance.

## 3. Results and Discussion

### 3.1. Preparation and Characterization of the CS NM

**Figure 1:**
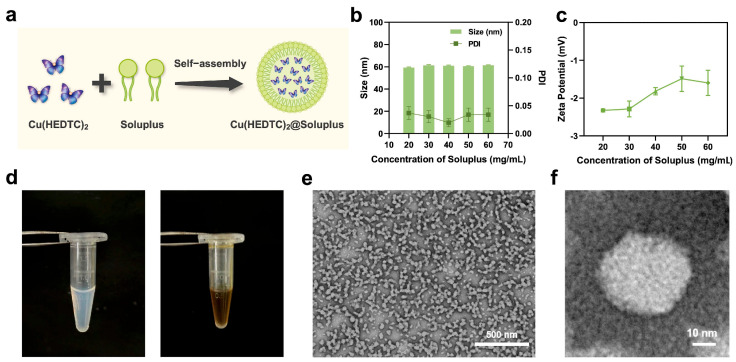
Formulation and morphology of CS NM. (**a**) Schematic illustration of the fabrication of CS NM. (**b**,**c**) Hydrodynamic size distribution, polydispersity index (PDI), and zeta potential of Soluplus nanomicelles at different concentrations (in ultrapure water) demonstrating optimal micelle formation at 40 mg/mL (*n* = 3). (**d**) Macroscopic appearance showing the enhanced solubility of Cu(HEDTC)_2_ after encapsulation in Soluplus nanomicelles (left: blank Soluplus nanomicelles; right: CS NM). (**e**,**f**) Representative transmission electron microscopy (TEM) images of CS NM (scale bars: 500 nm, 10 nm). Data are presented as mean ± SD.

CS NM was obtained by self-assembly of Cu(HEDTC)_2_ and Soluplus using a thin-film dispersion method, and the synthetic scheme is shown in Figure 1a. The Soluplus concentration for preparing CS NM was optimized by characterizing its particle size, polydispersity index (PDI), and zeta potential (Figure 1b,c). According to the PDI results, the concentration of Soluplus at 40 mg/mL had the smallest PDI and the most homogeneous particle size, at which concentration the CS NM prepared was obtained as shown in Figure 1d. Cu(HEDTC)_2_ is poorly soluble in water, but no precipitate was observed in the CS NM solution (Figure 1d), indicating that Soluplus contributed to enhancing the solubility of Cu(HEDTC)_2_ in water. The TEM image showed that the prepared CS NM had a uniform particle size and a spherical structure (Figure 1e,f), indicating well-formed nanostructural integrity. Notably, the PEGylated architecture of Soluplus has been extensively documented to confer steric stability through the formation of a hydrated protective layer that prevents micelle aggregation while enhancing electrostatic interactions [31]. Previous studies have demonstrated that Soluplus-based nanomicelles encapsulating fluorescent probes maintained their appearance, particle size, PDI, zeta potential, and fluorescence intensity essentially unchanged after 10 days of storage [32]. Although the stability of CS NM under physiological conditions requires further investigation, the inherent properties of Soluplus combined with the established literature evidence strongly suggest that CS NM possesses sufficient stability for subsequent experimental studies.

**Figure 2:**
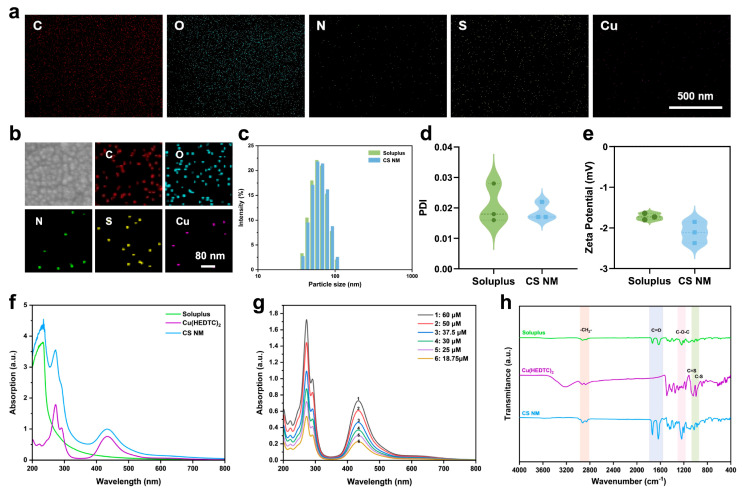
Spectral and physicochemical characterization of CS NM. (**a**,**b**) Representative elemental mapping (C, O, N, S, Cu) and scanning electron microscopy (SEM) images of CS NM verifying successful encapsulation of Cu(HEDTC)_2_ (scale bars: 500 nm, 80 nm; Cu(HEDTC)_2_ formula: C_10_H_20_CuN_2_O_4_S_4_). (**c**–**e**) Hydrodynamic size distribution, PDI, and zeta potential of Soluplus nanomicelles and CS NM (in ultrapure water) (*n* = 3). (**f**) UV–Vis spectra of Soluplus nanomicelles, Cu(HEDTC)_2_, and CS NM confirming Cu(HEDTC)_2_ encapsulation in CS NM through characteristic absorption peaks. (**g**) Concentration-dependent UV–Vis spectra of Cu(HEDTC)_2_ used for establishing the standard calibration curve of Cu(HEDTC)_2_ (*n* = 3). (**h**) Fourier transform infrared (FTIR) spectra of Soluplus nanomicelles, Cu(HEDTC)_2_, and CS NM demonstrating successful fabrication of CS NM. Data are presented as mean ± SD.

To confirm the encapsulation of hydrophobic Cu(HEDTC)_2_ within CS NM, elemental mapping analysis of the CS NM was performed, which revealed the distribution of Cu, N, and S, confirming the successful encapsulation of Cu(HEDTC)_2_ within the Soluplus nanomicelle (Figure 2a,b). The CS NM displayed a narrow size distribution, characterized by a hydrodynamic particle size of 61.34 ± 0.22 nm (Figure 2c) and a PDI of 0.019 ± 0.003 (Figure 2d). These results indicate a uniform and acceptable particle size distribution. Furthermore, the zeta potential of the CS NM was measured to be approximately −2.11 ± 0.26 mV (Figure 2e), which is regarded as nearly neutral-charged. The encapsulation of Cu(HEDTC)_2_ was further characterized by UV–Vis and FTIR analyses. UV–Vis spectral analysis of CS NM demonstrated increased absorptions at 273 nm and 434 nm compared to Soluplus, which were the characteristic absorption peaks for Cu(HEDTC)_2_, indicating the successful loading of Cu(HEDTC)_2_ (Figure 2f). In the UV–Vis spectroscopic analysis, the calibration curve for Cu(HEDTC)_2_ was determined with a coefficient of determination (*R*^2^) of 0.9997, and the encapsulation efficiency (EE %) of Cu(HEDTC)_2_ within the CS NM was calculated to be 89.90 ± 2.10% (Figure 2g; Table S1, Supporting Information; Figure S1, Supporting Information). These results indicated that Cu(HEDTC)_2_ was effectively encapsulated and retained within Soluplus self-assembled nanomicelles. Moreover, the FTIR spectrum provided further evidence of CS NM generation (Figure 2h). The FTIR spectrum of CS NM exhibited characteristic peaks at 2925 cm⁻^1^ and 2855 cm⁻^1^, corresponding to the asymmetric and symmetric stretching vibrations of CH_2_ groups in Soluplus alkyl chains [33], with overlapping peaks from the ethylene groups (-CH_2_-CH_2_-) of Cu(HEDTC)_2_, confirming the coexistence of both components in the CS NM. The sharp peaks shown at 1735 cm^−1^ and 1630 cm^−1^ were attributed to the stretching vibration of ester and the amide carbonyl groups (C=O) in Soluplus, respectively [34]. Another notable characteristic was the sharp peak at 1233 cm⁻^1^, which was typically correlated with the stretching vibrations of the ether linkage (C-O-C) within Soluplus, serving as a significant indicator confirming the existence of Soluplus [35]. Furthermore, the FTIR spectrum of CS NM displayed C=S and C-S stretching vibration bands of Cu(HEDTC)_2_ at 1030 cm^−1^ and 980 cm^−1^, respectively, demonstrating that Cu(HEDTC)_2_ was successfully loaded in the CS NM. Collectively, the above characterization results confirmed the successful fabrication of CS NM.

### 3.2. Efficient Delivery of Copper and Anti-Cancer Effect Using CS NM In Vitro

**Figure 3:**
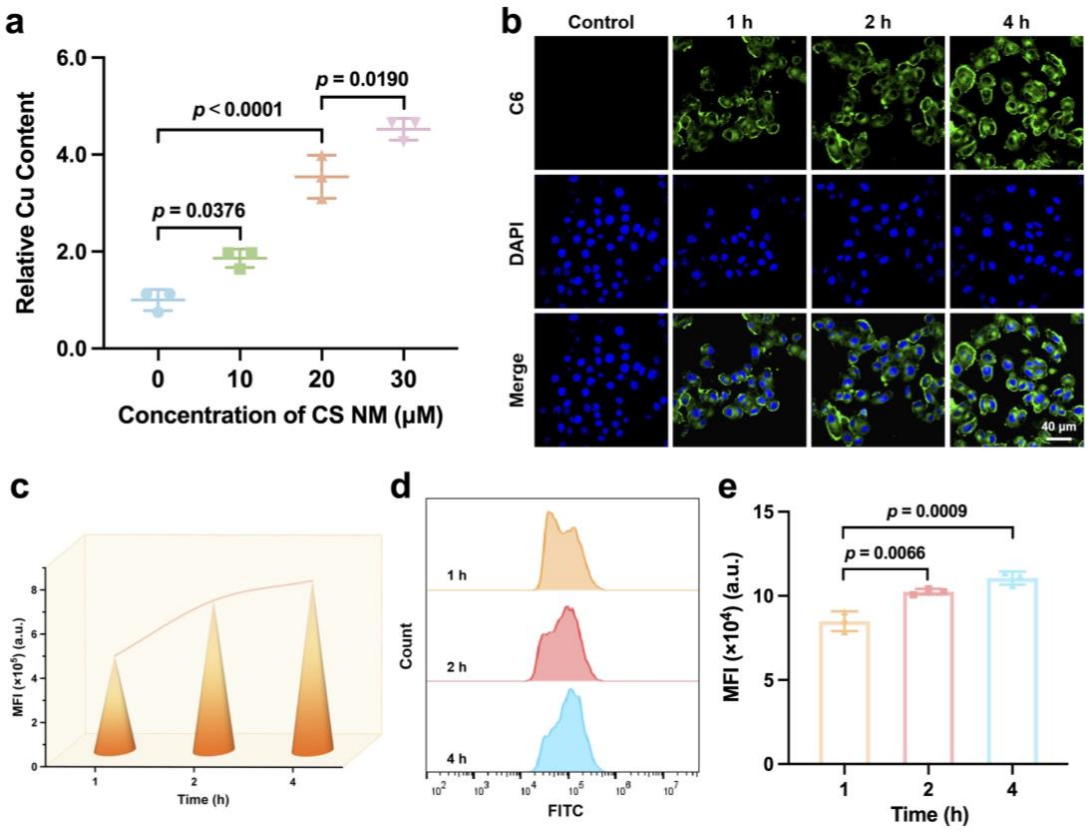
In vitro evaluation of copper delivery efficiency of CS NM in B16 cells. (**a**) Intracellular copper uptake in B16 cells after 4 h treatment with CS NM (0, 10, 20, and30 μM), measured by cell copper assay kit, and normalized to the total protein concentration determined by the BCA protein assay (*n* = 3). (**b**,**c**) Representative confocal laser scanning microscopy (CLSM) images and CLSM semi-quantitative analysis of B16 cells incubated with coumarin 6-labeled CS NM for 1, 2, and 4 h, respectively (green: coumarin 6-labeled CS NM, blue: DAPI; scale bar: 40 μm; *n* = 3). Fluorescence intensity was quantified using ImageJ (v1.53) and normalized to cell count. (**d**,**e**) Flow cytometry profiles and corresponding quantitative analysis of B16 cells treated with coumarin 6-labeled CS NM for 1, 2, and 4 h, respectively (*n* = 3). Fluorescence intensity was quantified using ImageJ (v1.53) and normalized to cell count. Data are presented as mean ± SD. *p* values in (**a**,**e**) were calculated by one-way ANOVA.

The increase in intracellular copper content was closely related to the occurrence of cuproptosis [13]. Considering the important role of copper in cuproptosis, we evaluated the intracellular copper level with mice melanoma B16 as a model cell. The intracellular copper level was visualized with a copper assay kit using different concentrations of CS NM (Figure 3a). The results showed that after 24 h incubation, the copper content in B16 cells treated with CS NM was significantly higher than that in the untreated group. Notably, the intracellular copper content gradually increased with rising concentrations of CS NM. The copper content in the B16 cells treated with 30 μM CS NM was 4.5 times higher than that in untreated cells. To further investigate the cellular uptake of CS NM in B16 cells, a fluorescent dye C6 was utilized to label CS NM, and the cellular uptake process was studied in a time-dependent manner using CLSM. CLSM images revealed that the green fluorescence signal observed inside the B16 cells gradually intensified as the incubation time was prolonged (Figure 3b). The fluorescence intensity in cells incubated for 4 h was 1.8 times higher than that observed after 1 h (Figure 3c), indicating that the cellular uptake process of CS NM was time-dependent. The cellular uptake process was further validated by flow cytometry. The results demonstrated that the internalization of CS NM by B16 cells gradually increased with prolonged incubation time, and the fluorescence intensity in cells incubated for 4 h was 1.3 times higher than after 1 h (Figure 3d,e). The enhanced cellular uptake efficiency can be attributed to the nanoparticle properties of CS NM that exploit the enhanced permeability and retention (EPR) effect. Extensive studies have demonstrated that nanoparticles within the 50–400 nm size range [36] and possessing negative surface charge [37] preferentially accumulate in tumor tissues through the leaky tumor vasculature. The CS NM exhibits optimal physicochemical characteristics for EPR effect, including a hydrodynamic particle size of 61.34 ± 0.22 nm and a zeta potential of −2.11 ± 0.26 mV, which are well aligned with the established EPR-favorable parameters for nanocarriers. These features collectively contribute to the enhanced cellular copper-uptake efficiency observed in our study. Due to the scope of this research, in vivo biodistribution studies to directly validate the EPR effect are yet to be conducted; they will be a key focus of subsequent animal experiments. However, we recognize that this copper delivery strategy may pose risks of systemic copper overload, a critical issue that must be addressed for the clinical translation of cuproptosis therapy. The EPR effect of CS NM facilitates its selective accumulation in tumor tissues, thereby mitigating systemic copper overload risks. Nevertheless, we acknowledge that definitive conclusions require further validation through in vivo animal studies to ensure the safety profile for potential clinical application. Collectively, these results suggested that CS NM functioned as a nanoscale shuttle to efficiently transport extracellular copper into B16 cells, significantly enhancing intracellular copper levels and promoting robust cuproptosis induction.

**Figure 4:**
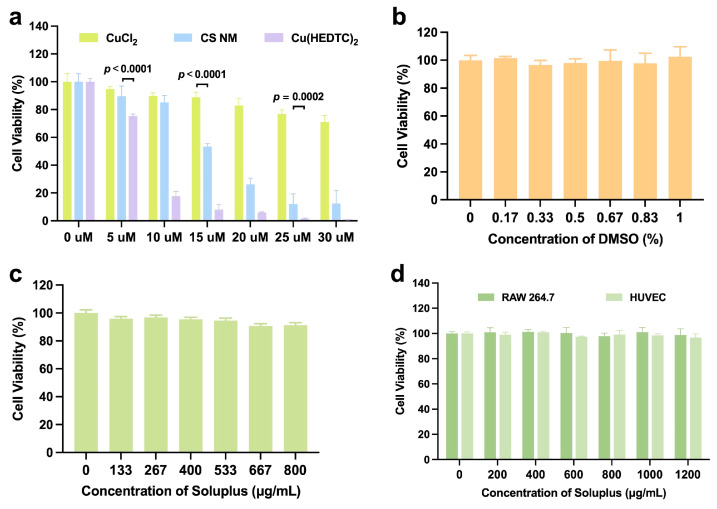
In vitro evaluation of antitumor efficacy and biocompatibility of CS NM. (**a**) Comparative cytotoxicity of B16 cells after 24 h incubation with varying concentrations of CuCl_2_, CS NM, and Cu(HEDTC)_2_ (0, 5, 10, 15, 20, 25, and30 μM, Cu-equivalent concentration), respectively. B16 cell viability was evaluated using CCK-8 assay (*n* = 6). (**b**) Viability of B16 cells treated with DMSO solvent for 24 h (0, 017, 0.33, 0.5, 0.67, 0.83, and 1% *v*/*v*, corresponding to Cu(HEDTC)_2_ concentrations in panel (**a**)). B16 cell viability was evaluated using CCK-8 assay (*n* = 6). (**c**) Viability of B16 cells treated with Soluplus nanomicelles for 24 h (0, 133, 267, 400, 533, 667, and 800 μg/mL, corresponding to CS NM concentrations in panel (**a**)). B16 cell viability was evaluated using CCK-8 assay (*n* = 6). (**d**) Biocompatibility of Soluplus in RAW 264.7 and HUVECs after 24 h treatment (0, 200, 400, 600, 800, 1000, and 1200 μM). Viability of RAW 264.7 and HUVECs were evaluated using CCK-8 assay (*n* = 6). Data are presented as mean ± SD. *p* values in (**a**) were calculated by two-way ANOVA.

After confirming the superior copper delivery efficiency of CS NM, we proceeded to evaluate its cytotoxic effect using the CCK-8 assay. We examined the cytotoxicity of a range of CS NM concentrations on B16 cells. The results demonstrated that CS NM exhibited potent cytotoxicity against B16 cells (IC_50_: 14.12 μM) (Figure S2, Supporting Information), and the cell-killing efficiency of the CS NM treatment was dose-dependent (Figure 4a). Notably, as expected, when B16 cells were treated with CS NM at concentrations greater than 10 μM, the cytotoxicity was significantly superior to that of CuCl_2_ (*p* < 0.0001) (Figure 4a). This difference can be attributed to the efficient copper delivery mediated by the nanomicellar system. The hydrophilic shell of Soluplus micelles could facilitate cellular uptake [38], thereby substantially improving intracellular bioavailability. Importantly, the substantially higher cytotoxicity of both CS NM and free Cu(HEDTC)_2_ compared to CuCl_2_ (*p* < 0.0001) underscored the critical role of the copper ionophore in enhancing cellular copper delivery (Figure 4a). Interestingly, CS NM showed reduced cytotoxicity relative to free Cu(HEDTC)2 (*p* < 0.0001) (Figure 4a). Free Cu(HEDTC)_2_ could rapidly penetrate the cell membrane to exert acute toxicity. In contrast, CS NM required the dissociation of the Soluplus matrix to release the active ingredient, Cu(HEDTC)_2_, resulting in a relatively lower cell-killing efficiency compared to free Cu(HEDTC)_2_ in a limited timeframe.

Comprehensive safety evaluations confirmed that neither DMSO (0–1% v/v, matching Cu(HEDTC)_2_ solvent concentrations) nor Soluplus (0–800 μg/mL, equivalent to CS NM doses) caused significant cytotoxicity in B16 cells (above 90% cell viability) (Figure 4b,c), confirming that the observed antitumor effects specifically derived from Cu(HEDTC)_2_ pharmacological activity rather than excipient toxicity. Furthermore, we assessed the biocompatibility of Soluplus with RAW 264.7 cells (a murine macrophage cell line) and HUVECs (a human umbilical vein endothelial cell line) using the CCK-8 assay. The results demonstrated above 95% cell viability across even high concentration ranges (0–1200 μM) after 24 h exposure (Figure 4d), fully confirming the excellent biocompatibility of this blank nanoshuttle within the experimental dosage range. These findings indicate that CS NM could induce a tumor-killing effect against B16 cells in vitro.

### 3.3. Evaluation of Oxidative Stress Induced by CS NM In Vitro

**Figure 5:**
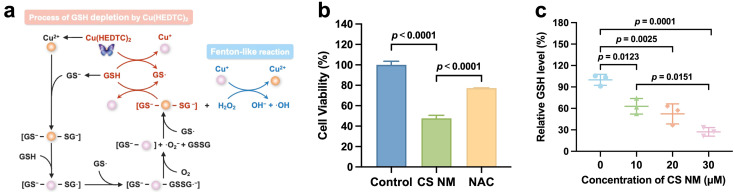
Evaluation of GSH-scavenging effect of CS NM in B16 cells in vitro. (**a**) Schematic illustration of the oxidative stress induction process by CS NM in B16 cells. (**b**) Cell viability of B16 cells after different treatments (untreated control, 15 μM CS NM, and 15 μM CS NM plus 50 μM NAC), normalized to untreated controls (set as 100%) (*n* = 3). (**c**) Relative intracellular GSH levels measured by GSH level assay kit in B16 cells after 24 h incubation with different concentrations of CS NM (0, 10, 20, and 30 μM), normalized to the total protein concentration determined by the BCA protein assay (*n* = 3). Data are presented as mean ± SD. Statistical significance was determined by one-way ANOVA.

Once they have entered the tumor cells, copper ions can bind to glutathione (GSH), which is overexpressed within tumor cells [29]. During this process, intracellular GSH, acting as a thiol-containing copper chelator, can inhibit cuproptosis. Therefore, the reduction of intracellular GSH concentration is conducive to enhancing the cuproptosis of cells [39]. As illustrated in Figure 5a, copper ions can directly bind to GSH, thereby effectively depleting free GSH in tumor cells [40]. Moreover, copper ions could also induce ROS generation via a Fenton-like reaction [41]. The combined effect of antioxidant GSH depletion and ROS accumulation disrupts cellular redox homeostasis, ultimately triggering tumor cell death. Given the presence of copper ions in Cu(HEDTC)_2_ structures, we hypothesized that CS NM might induce cuproptosis through Cu(HEDTC)_2_-mediated oxidative stress. To validate this mechanism, we first treated B16 cells with the antioxidant N-acetyl-L-Cysteine (NAC) alongside CS NM. Strikingly, 50 μM NAC co-treatment significantly rescued CS NM-induced cytotoxicity, increasing cell viability from 47.62 ± 2.91% to 77.24 ± 0.3% (*p* < 0.001) (Figure 5b). These results conclusively demonstrate the pivotal role of oxidative stress in CS NM’s antitumor effects.

Subsequently, to demonstrate the GSH scavenging ability of CS NM, GSH level assay kit was used to detect the change in GSH content in B16 cells treated with varying concentrations of CS NM. Quantitative analysis revealed a significant decrease in intracellular GSH level in CS NM-treated groups compared to the control group, with a dose-dependent decline observed (Figure 5c). Specifically, treatment with CS NM at a concentration of 10 μM led to a notable reduction in GSH content, and when the concentration was increased to 30 μM, intracellular GSH level dropped to approximately 27% of that in untreated cells. It was worth noting that although CS NM consumption led to a depletion of copper ions during GSH scavenging, overall intracellular copper content was found to increase based on cellular copper uptake results (Figure 3a). These findings collectively indicated that CS NM exhibits biological activity in scavenging GSH, which might facilitate the occurrence of cuproptosis.

**Figure 6:**
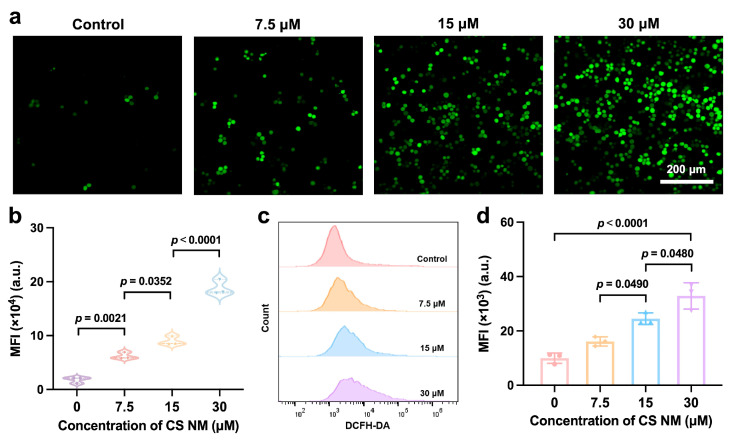
Evaluation of ROS generated by CS NM in B16 cells in vitro. (**a**,**b**) Representative confocal laser scanning microscopy (CLSM) images and semi-quantitative analysis of B16 cells stained with DCFH-DA (green: ROS) after 4 h treatment with varying CS NM concentrations (0, 7.5, 15, and 30 μM), showing ROS elevation (scale bar: 200 μm; *n* = 3). Fluorescence intensity was quantified using ImageJ (v1.53) and normalized to cell count. (**c**,**d**) Flow cytometry profiles and corresponding quantitative analysis of B16 cells stained with DCFH-DA after 4 h exposure to different CS NM concentrations (0, 7.5, 15, and 30μM) (*n* = 3). Fluorescence intensity was quantified using ImageJ (v1.53) and normalized to cell count. Data are presented as mean ± SD. Statistical significance was determined by one-way ANOVA.

As shown in Figure 5a, copper ions could also induce the generation of reactive oxygen species (ROS) via a Fenton-like reaction [41]. To evaluate the ROS production in B16 cells after CS NM treatment, a fluorescent probe 2,7-dichlorodihydrofluorescein diacetate (DCFH-DA) was employed as a detector to study intracellular ROS levels. CLSM images revealed that control group cells exhibited weak fluorescent signals, indicating low ROS levels. In contrast, the CS NM-treated group began to show noticeable green fluorescence signals when the CS NM concentration was 7.5 μM, and the fluorescence intensity significantly increased with rising CS NM concentrations (Figure 6a,b). Flow cytometry further confirmed ROS generation. The results demonstrated that ROS content in B16 cells gradually increased with rising CS NM concentrations, and the fluorescence intensity in cells treated with 30 μM CS NM was 3.3 times higher than that of the control group (Figure 6c,d), suggesting that copper ions induce ROS production in B16 cells through a Fenton-like reaction. Notably, as one of the most crucial intracellular antioxidants, the depletion of GSH directly compromises cellular ROS scavenging capacity, further promoting ROS generation and oxidative stress [42]. These results indicate that CS NM, by depleting GSH and mediating Fenton-like reactions, induce a substantial increase in ROS production. This oxidative stress amplification critically contributed to cuproptosis induction in B16 cells.

### 3.4. CS NM Induced Cuproptosis in B16 Cells

**Figure 7:**
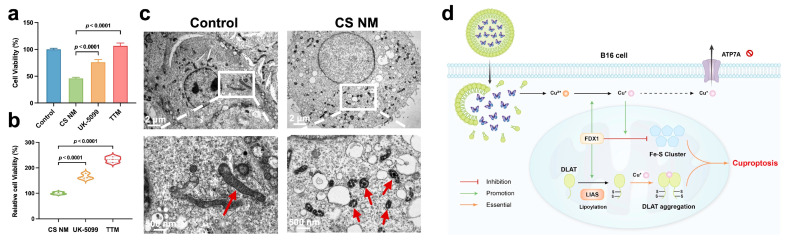
Functional validation of cuproptosis induced by CS NM in B16 cells in vitro. (**a**) Cell viability of B16 cells after different treatments (untreated control, 15 μM CS NM, 15 μM CS NM plus 10 μM UK-5099, and 15 μM CS NM plus 10 μM TTM), normalized to untreated controls (set as 100%) (*n* = 3). (**b**) Relative viability of B16 cells treated with 15 μM CS NM plus cuproptosis inhibitors (10 μM UK-5099, 10 μM TTM) for 24 h (*n* = 3). (**c**) Representative Bio-TEM images showing mitochondrial ultrastructural changes in B16 cells after 24 h treatment with 15 μM CS NM, including shrinkage, increased membrane density, reduced cristae (scale bars: 2 μm, 500 nm). (**d**) Schematic illustration of CS NM-induced cuproptosis. Data are presented as mean ± SD. Statistical significance was determined by one-way ANOVA.

Given the exceptional copper-delivery capacity, GSH depletion, and ROS generation properties of CS NM, we hypothesized that CS NM could effectively trigger cuproptosis in B16 cells. To verify whether the cytotoxic effects of CS NM were indeed mediated by cuproptosis, we pretreated B16 cells with two classical cuproptosis inhibitors, UK-5099 and TTM, and measured the relative cell viability (Figure 7a,b). The results demonstrated that CS NM treatment alone significantly induced B16 cell death, whereas pre-treatment with UK-5099 or TTM markedly attenuated CS NM-induced cell death, indicating that the cytotoxic effect of CS NM was dependent on cuproptosis and would be reversed by cuproptosis inhibitors.

Cuproptosis has been demonstrated to be closely associated with the aberrant aggregation of mitochondrial lipoylated proteins, a process that is accompanied by significant mitochondrial dysfunction [1]. During this process, mitochondria exhibit characteristic morphological alterations, including mitochondrial shrinkage and reduction or even the disappearance of cristae [43]. In subsequent studies, we employed Bio-TEM to visualize mitochondrial morphological changes in cells treated with CS NM. The Bio-TEM images clearly revealed distinct alterations in mitochondrial morphology (Figure 7c). In control group cells, mitochondria displayed regular elliptical or elongated structures with well-defined cristae structures. In contrast, mitochondria in B16 cells treated with CS NM exhibited shrinkage, increased membrane density, reduced cristae, and even vacuolization, indicating severe mitochondrial dysfunction. These findings collectively indicated that CS NM induced cuproptosis in B16 cells.

**Figure 8:**
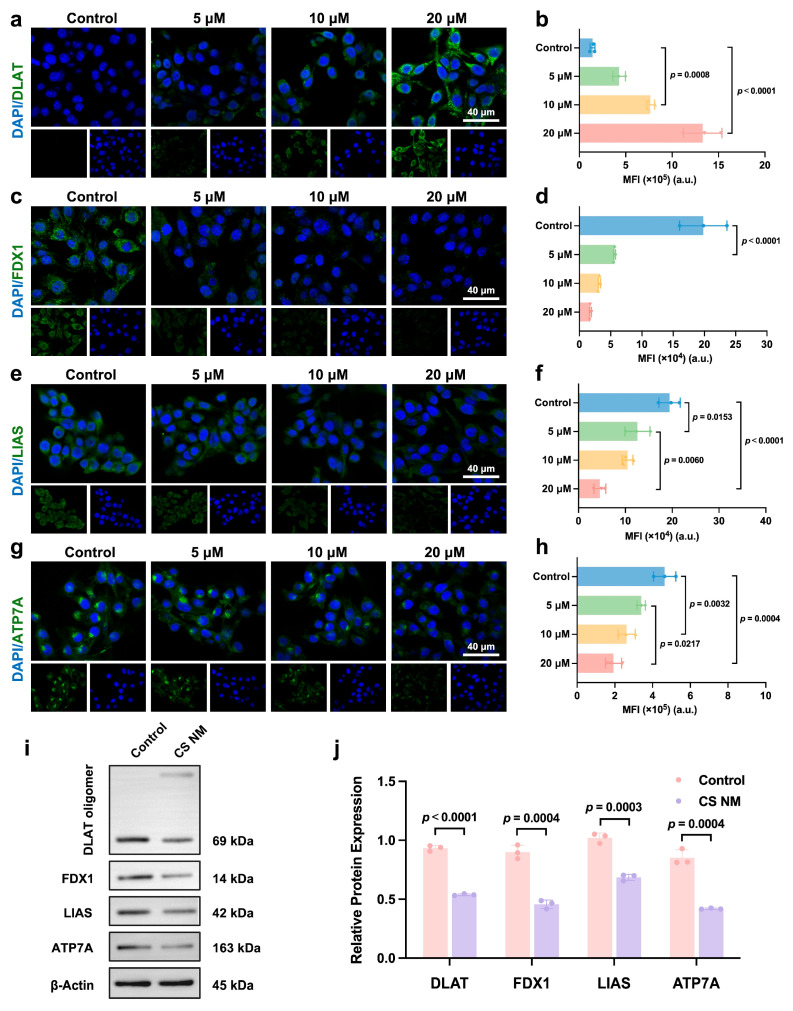
Validation of CS NM-induced cuproptosis mechanisms. (**a**,**b**) Representative immunofluorescence images (green: DLAT oligomer) and semi-quantitative analysis of DLAT expression in B16 cells after 24 h treatment with different CS NM concentrations (0, 5, 10, and 20 μM) (scale bar: 40 μm; *n* = 3, analyzed by ImageJ v1.53). (**c**,**d**) Representative immunofluorescence images (green: FDX1) and semi-quantitative analysis of FDX1 expression in B16 cells after 24 h treatment with different CS NM concentrations (0, 5, 10, and 20 μM) (scale bar: 40 μm; *n* = 3, analyzed by ImageJ v1.53). (**e**,**f**) Representative immunofluorescence images (green: LIAS) and semi-quantitative analysis of LIAS expression in B16 cells after 24 h treatment with different CS NM concentrations (0, 5, 10, and 20 μM) (scale bar: 40 μm; *n* = 3, analyzed by ImageJ v1.53). (**g**,**h**) Representative immunofluorescence images (green: ATP7A) and semi-quantitative analysis of ATP7A expression in B16 cells after 24 h treatment with different CS NM concentrations (0, 5, 10, and 20 μM) (scale bar: 40 μm; *n* = 3, analyzed by ImageJ v1.53). (**i**,**j**) Western blot analysis and semi-quantification of DLAT, FDX1, LIAS, and ATP7A levels in B16 cells after CS NM treatment (*n* = 3). Data are presented as mean ± SD. *p* values in (**b**,**d**,**f**,**h**) were calculated by one-way ANOVA, while those in (**j**) were determined by unpaired Student’s two-sided *t*-test. The original western blots can be found at Figures S3–S7.

As illustrated in Figure 7d, CS NM efficiently delivers copper ions into intracellular compartments. The released copper ions subsequently bind to DLAT, inducing toxic aggregation of DLAT and downregulating the expression of Fe-S cluster proteins such as Fe–S cluster proteins ferredoxin (FDX1), lipoic acid synthase (LIAS) and the copper transporter ATP7A, thereby triggering cuproptosis [3]. To further investigate the mechanisms of CS NM-induced cuproptosis, we visualized the oligomerization of DLAT and changes in the expression levels of FDX1, LIAS, and ATP7A using immunofluorescence. Immunofluorescence imaging shows that DLAT without detectable aggregation was observed in B16 cells untreated with CS NM (Figure 8a,b). In contrast, CS NM treatment induced pronounced DLAT aggregation in a dose-dependent manner, confirming CS NM can induce DLAT aggregation. Furthermore, the green fluorescence signals corresponding to FDX1, LIAS, and ATP7A were significantly attenuated in CS NM-treated cells (Figure 8c–h), suggesting substantial downregulation of these proteins. Subsequent Western blot analysis of protein extracts from CS NM-treated B16 cells corroborated these findings (Figure 8i,j). Compared with the control group, CS NM significantly reduced the expression levels of FDX1, LIAS, and ATP7A in B16 cells, providing conclusive evidence that CS NM induced cuproptosis in B16 cells. It can be collectively demonstrated that CS NM induced mitochondrial copper overload, triggering abnormal aggregation of DLAT and downregulation of FDX1, LIAS, and ATP7A proteins. This cascade of events ultimately disrupted mitochondrial structure and function, leading to cuproptosis in B16 cells.

## 4. Conclusions

In summary, we have pioneered the discovery and validation of the unique role of Cu(HEDTC)_2_ as a novel copper ionophore in inducing cuproptosis. In this study, we constructed a “Winged Cu” nanoshuttle system by encapsulating Cu(HEDTC)_2_ within Soluplus to develop a CS NM nanosystem, which effectively induced cuproptosis in B16 model cells by disrupting intracellular copper homeostasis. In vitro experiments demonstrated that CS NM functioned as a nanoshuttle, significantly enhancing copper level in B16 cells and promoting substantial intracellular copper accumulation. Furthermore, CS NM effectively induced cuproptosis in B16 cells. Bio-TEM analysis further confirmed characteristic cuproptosis-associated mitochondrial damage in CS NM-treated B16 cells, including mitochondrial shrinkage and cristae disruption. These findings not only establish, for the first time, the application potential of Cu(HEDTC)_2_ as an efficient copper ionophore for inducing cuproptosis, but also provide a novel theoretical foundation for developing cuproptosis-based antitumor therapeutic strategies.

While our in vitro results demonstrate CS NM’s ability to induce cuproptosis in B16 cells, the absence of in vivo data represent a key limitation. Without animal studies, we cannot assess critical translational aspects including the therapeutic efficacy, biodistribution, and biosafety. Therefore, in our ongoing studies, we are scheduled to perform in-depth in vivo studies to pave the way for preclinical/clinical trials. Additionally, we fully recognize the scientific importance of conducting comparative experiments with elesclomol and disulfiram to further validate the advantages of our CS NM system. However, due to current limitations in research funding and timeline constraints, we were unable to include these critical comparisons in the present study. Moving forward, we have prioritized these comparative studies in our ongoing research plan to establish CS NM’s potential for clinical translation. It is particularly noteworthy that although this study primarily focuses on melanoma as a representative malignant tumor, the design principles of the “Winged Cu” nanoshuttle system and the elucidated mechanisms of cuproptosis induction established herein are expected to provide crucial references for developing novel copper ionophore-loaded nanosystems to target other malignancies.

## Figures and Tables

**Table 1:** Comparative analysis of current copper delivery systems and CS NM.

Category	Limitations of Previous Studies	Distinctiveness of CS NM	Reference
Material	Limited investigational materials (copper ionophores such as elesclomol and disulfiram are commonly used in studies)	Cu(HEDTC)_2_ was proposed for the first time as a novel copper ionophore for cancer therapy	[8,10,11]
Copper delivery efficiency	Low copper delivery efficiency (upon elesclomol treatment, intracellular copper levels increased only approximately 2-fold compared to controls)	Significantly enhanced copper delivery efficiency (CS NM elevated intracellular copper levels approximately 5-fold compared to controls)	[23]
Cytotoxicity	Low cytotoxicity (high IC_50_ values, IC_50_ > 70 μM)	CS NM enhanced cytotoxicity (IC_50_: 14.12 μM)	[24,25]

## Data Availability

The original contributions presented in this study are included in the article and further inquiries can be directed to the corresponding authors.

## Supplementary Materials

The following supporting information can be downloaded at: https://www.mdpi.com/article/10.3390/biom15060895/s1, Figure S1: Standard calibration of Cu(HEDTC)_2_. Figure S2: Dose–response curves for B16 cells treated with Cu(HEDTC)_2_. Table S1. UV–Vis absorbance measurements of Cu(HEDTC)_2_ at different concentrations. Table S2. Standard deviations, confidence intervals, and *p*-values for all quantitative data.

## Abbreviations

The following abbreviations are used in this manuscript:

CS NM	Cu(HEDTC)_2_@Soluplus nanomicelle
TCA	Tricarboxylic acid
DLAT	Dihydrolipoamide S-acetyltransferase
Fe-S	Iron–sulfur
DSF	Disulfiram
Cu(HEDTC)_2_	Bis(2-hydroxyethyl)dithiocarbamic acid copper(II)
PEG	Polyethylene glycol
PVCL	Polyvinyl caprolactam
PVAc	Polyvinyl acetate
EPR	Enhanced permeability and retention
GSH	Glutathione
ROS	Reactive oxygen species
