# Supplementary material for: A Novel Copper Ionophore Nanoshuttle (Winged Cu) for Inducing Cuproptosis in B16 Melanoma Cells
Source: Biomolecules. 2025 Jun 18;15(6):895. doi: 10.3390/biom15060895 (PMC12190664; doi:10.3390/biom15060895)
Supplement: Supplementary file 1 [file biomolecules-15-00895-s001.zip › biomolecules-3668119-supplementary.pdf]

biomolecules

MDPI

Winged Cu: A Novel Copper Ionophore Nanoshuttle Triggering Cuproptosis

Yuhuan Wu, Ziyao Chang, Wenhao Wang, Chuanbin Wu, Xin Pan and Zhengwei Huang

Supplementary Data

Table S1. UV-Vis absorbance measurements of $Cu(HEDTC)_2$ at different concentrations.

Concentration (μM)	Absorbance (λ =434 nm)			Mean ± SD
	Replicate 1	Replicate 2	Replicate 3	
**18.75**	0.225	0.227	0.224	0.225 ± 0.002
25	0.302	0.305	0.301	0.303 ± 0.002
30	0.368	0.369	0.366	0.368 ± 0.002
37.5	0.461	0.462	0.460	0.461 ± 0.001
50	0.607	0.607	0.606	0.607 ± 0.001
60	0.727	0.728	0.725	0.727 ± 0.002

Table S2. Standard deviations, confidence intervals, and p-values for all quantitative data.

Experiment	Group	Mean	Std. Deviation	95% Confidence Interval		*p*-value
				Lower	Upper	
Characterization (Size)	Soluplus	60.71	0.26	60.08	61.34	-
	CS NM	61.34	0.22	60.80	61.88	-
Characterization (PDI)	Soluplus	0.02	0.01	0.00	0.04	-
	CS NM	0.02	0.00	0.01	0.03	-
Characterization (Zeta potential)	Soluplus	-1.72	0.08	-1.92	-1.52	-
	CS NM	-2.11	0.26	-2.76	-1.46	-
Intracellular Cu content	Control	1.00	0.22	0.45	1.55	-
	CS NM (10 μM)	1.86	0.19	1.39	2.34	0.0376 (vs. Control)
	CS NM (20 μM)	3.54	0.45	2.44	4.65	< 0.0001 (vs. Control)
	CS NM (30 μM)	4.52	0.22	3.96	5.08	< 0.0001 (vs. Control)
Cellular uptake	1 h	8.50	0.59	7.03	9.96	-
	2 h	10.24	0.17	9.82	10.66	0.0066 (vs. 1 h)
	4 h	11.06	0.39	10.09	12.02	0.0009 (vs. 1 h)
Cytotoxicity	CuCl2 (15 μM)	88.88	3.61	85.10	92.67	-
	CS NM (15 μM)	53.34	2.12	51.12	55.56	< 0.0001 (vs. Control)
	$Cu(HEDTC)_2$ (15 μM)	7.98	3.70	4.10	11.87	< 0.0001 (vs. Control)
Antioxidant Rescue	Control	100.00	3.54	91.20	108.80	< 0.0001 (vs. CS NM)
	CS NM	47.62	2.91	40.40	54.84	-
	NAC	77.24	0.30	76.48	78.00	< 0.0001 (vs. CS NM)
Intracellular GSH level	Control	100.00	7.62	81.07	118.90	-
	CS NM (10 μM)	62.97	10.89	35.92	90.02	0.0123 (vs. Control)
	CS NM (20 μM)	52.38	14.05	17.48	87.28	0.0025 (vs. Control)
	CS NM (30 μM)	27.20	6.01	12.27	42.13	0.0001 (vs. Control)
Intracellular ROS generation	Control	9.99	1.90	5.26	14.71	-
	CS NM (7.5 μM)	16.14	1.70	11.92	20.37	0.1845 (vs. Control)
	CS NM (15 μM)	24.50	2.13	19.20	29.79	0.0018 (vs. Control)
	CS NM (30 μM)	32.89	4.85	20.83	44.94	< 0.0001 (vs. Control)

Cell death	Control	100.00	2.18	94.59	105.40	< 0.0001 (vs. CS NM)
	CS NM	45.88	2.09	40.69	51.08	-
	UK-5099	75.92	5.18	63.04	88.80	< 0.0001 (vs. CS NM)
	TTM	106.60	5.24	93.55	119.60	< 0.0001 (vs. CS NM)
Immunofluorescence (DLAT oligomerization)	Control	1.42	0.27	0.75	2.10	-
	CS NM (5 μM)	4.29	0.68	2.60	5.97	0.0813 (vs. Control)
	CS NM (10 μM)	7.66	0.45	6.55	8.77	0.0008 (vs. Control)
	CS NM (20 μM)	13.30	2.07	8.15	18.45	< 0.0001 (vs. Control)
Immunofluorescence (FDX1)	Control	19.82	3.79	10.42	29.23	-
	CS NM (5 μM)	5.62	0.15	5.25	5.99	< 0.0001 (vs. Control)
	CS NM (10 μM)	3.19	0.19	2.71	3.67	< 0.0001 (vs. Control)
	CS NM (20 μM)	1.76	0.15	1.38	2.14	< 0.0001 (vs. Control)
Immunofluorescence (LIAS)	Control	19.45	2.26	13.83	25.06	-
	CS NM (5 μM)	12.58	2.68	5.94	19.23	0.0153 (vs. Control)
	CS NM (10 μM)	10.47	1.15	7.61	13.33	0.0029 (vs. Control)
	CS NM (20 μM)	4.57	1.27	1.41	7.73	< 0.0001 (vs. Control)
Immunofluorescence (ATP7A)	Control	4.64	0.59	3.17	6.11	-
	CS NM (5 μM)	3.40	0.22	2.86	3.94	0.0529 (vs. Control)
	CS NM (10 μM)	2.62	0.46	1.49	3.76	0.0032 (vs. Control)
	CS NM (20 μM)	1.93	0.43	0.87	2.99	0.0004 (vs. Control)
Western blot (DLAT oligomerization)	Control	0.93	0.02	0.87	0.99	-
	CS NM	0.54	0.01	0.52	0.56	< 0.0001 (vs. Control)
Western blot (FDX1)	Control	0.90	0.06	0.76	1.04	-
	CS NM	0.46	0.04	0.37	0.55	0.0004 (vs. Control)
Western blot (LIAS)	Control	1.02	0.04	0.92	1.12	-
	CS NM	0.69	0.03	0.62	0.75	0.0003 (vs. Control)
Western blot (ATP7A)	Control	0.85	0.07	0.69	1.02	-
	CS NM	0.42	0.01	0.41	0.43	0.0004 (vs. Control)

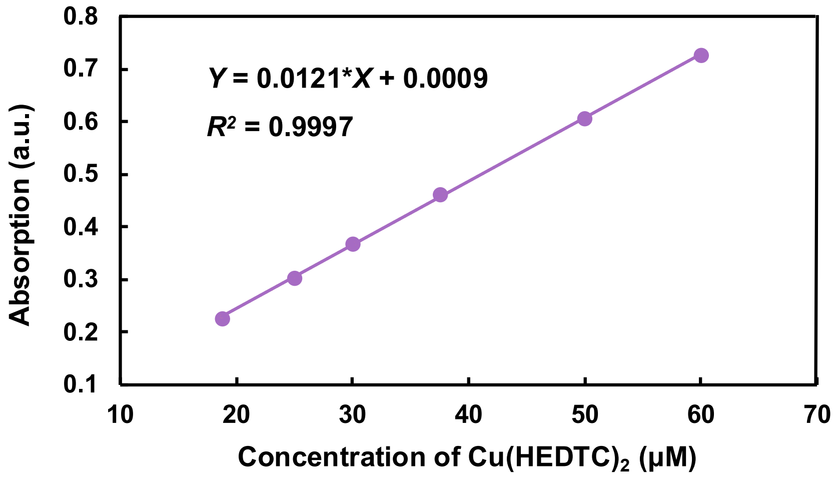

Figure S1. Standard calibration of $Cu(HEDTC)_2$.

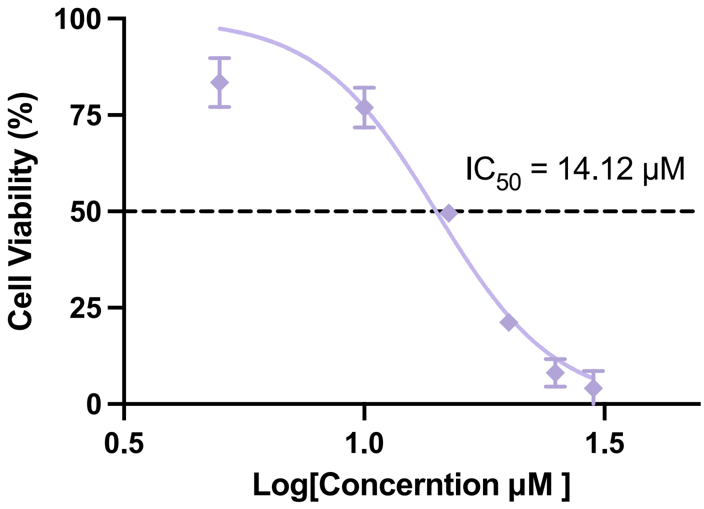

Figure S2. Dose–response curves for B16 cells treated with Cu(HEDTC)2.

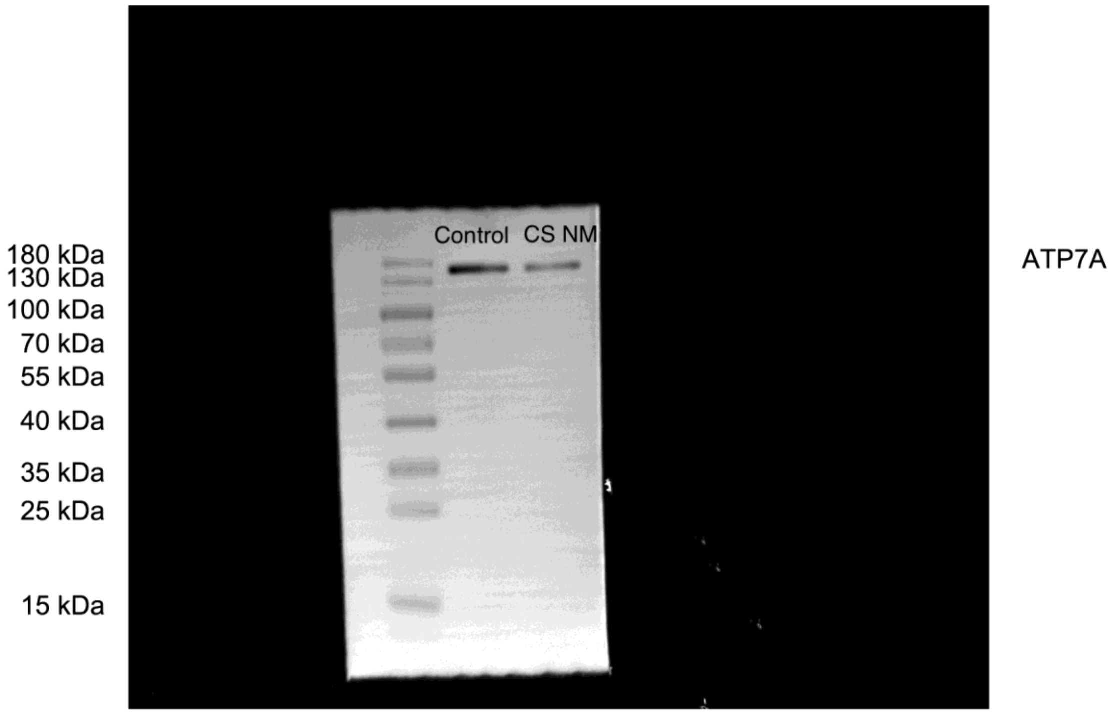

Figure S3. Original Western blot image-ATP7A

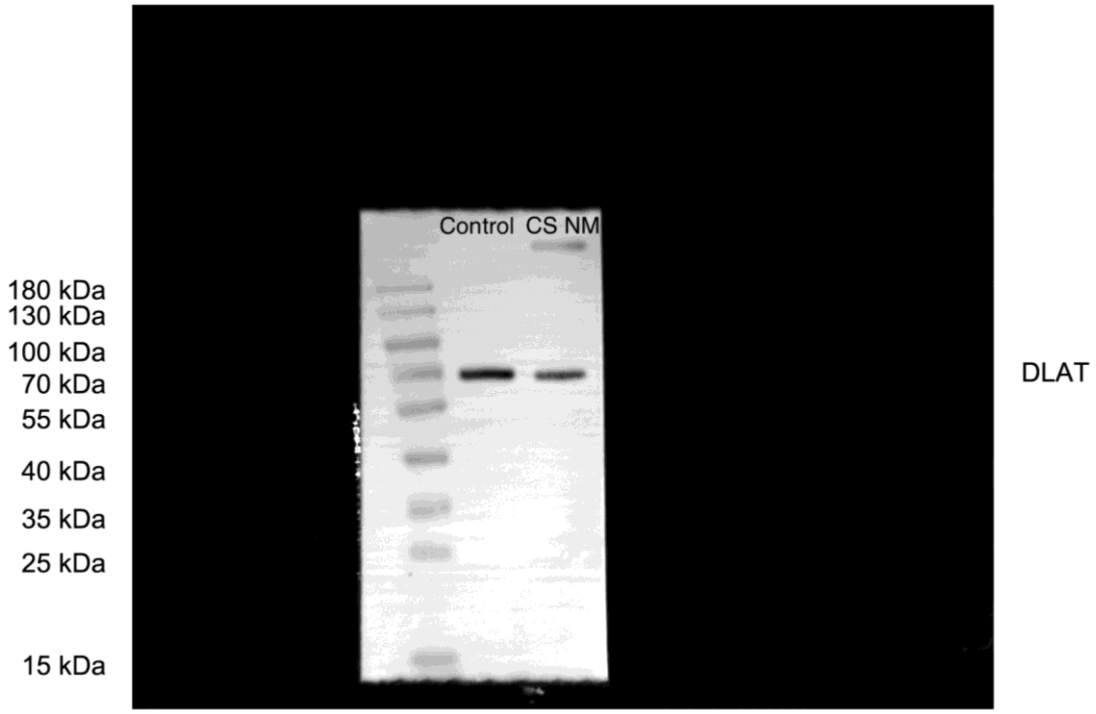

Figure S4. Original Western blot image-DLAT.

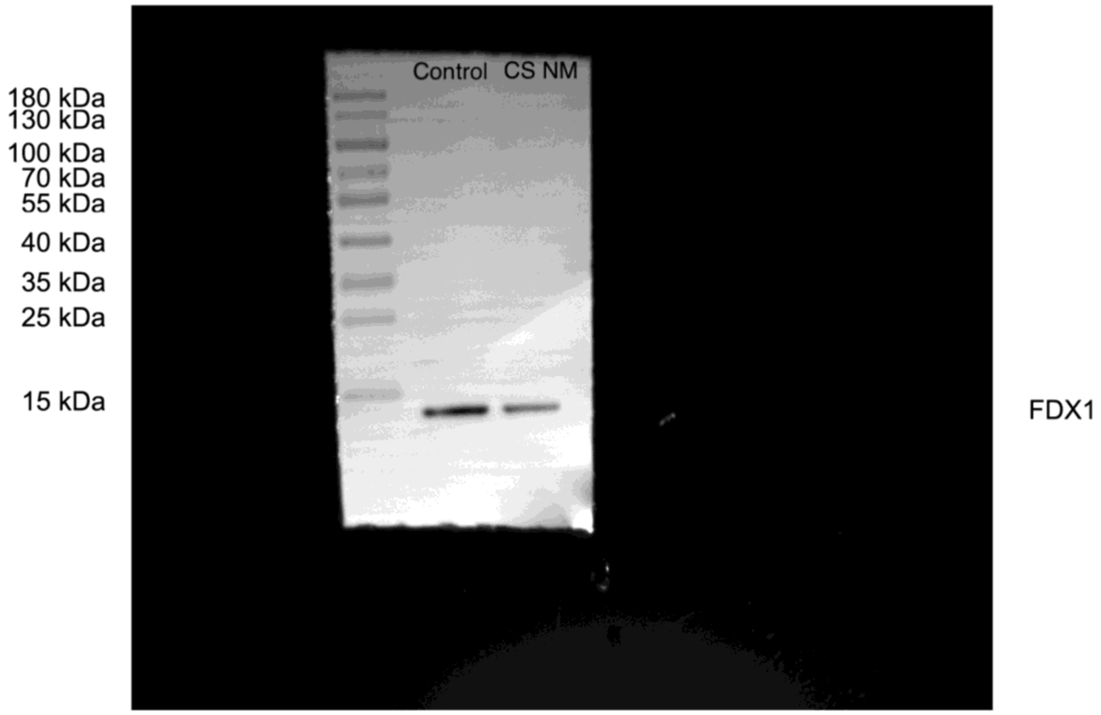

Figure S5. Original Western blot image-FDX1.

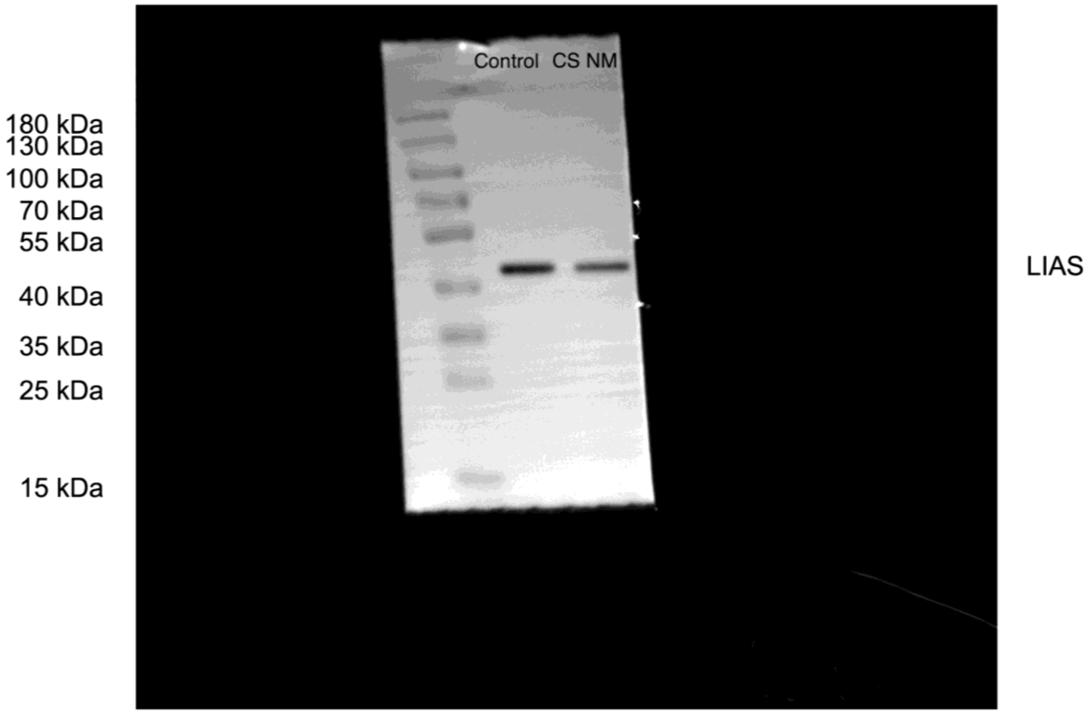

Figure S6. Original Western blot image-LIAS.

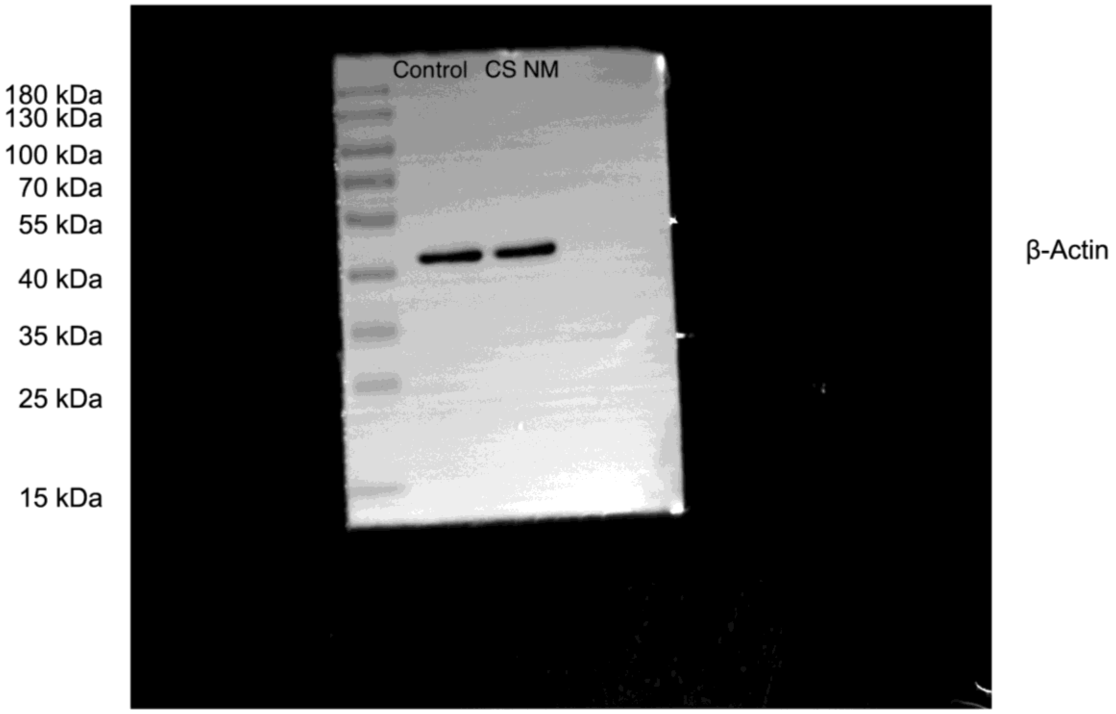

Figure S7. Original Western blot image-β-Actin.
